# ERA Registry Figure of the month Expected remaining years of life for dialysis and kidney transplant patients

**DOI:** 10.1093/ckj/sfaf091

**Published:** 2025-04-23

**Authors:** Vianda S Stel, Alberto Ortiz, Anneke Kramer

**Affiliations:** ERA Registry, Department of Medical Informatics, Amsterdam UMC—Location University of Amsterdam, Amsterdam, the Netherlands; Amsterdam Public Health Research Institute, Quality of Care, Amsterdam, the Netherlands; Department of Nephrology and Hypertension, IIS-Fundacion Jimenez Diaz UAM, Madrid, Spain; Department of Medicine, Universidad Autonoma de Madrid, Madrid, Spain; ERA Registry, Department of Medical Informatics, Amsterdam UMC—Location University of Amsterdam, Amsterdam, the Netherlands; Amsterdam Public Health Research Institute, Quality of Care, Amsterdam, the Netherlands

**Figure 1:**
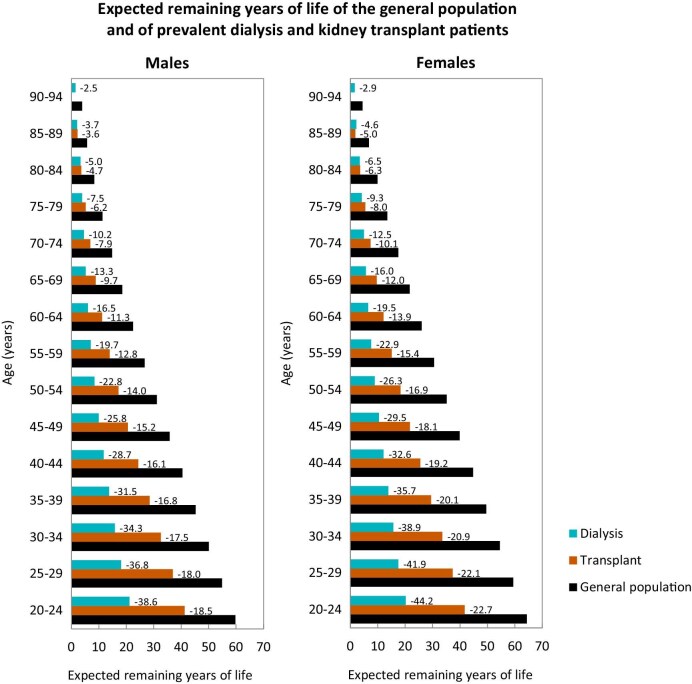
Expected remaining years of life in the general population and for prevalent dialysis and kidney transplant patients, for males (left panel) and females (right panel), by age group, using data from the 2018–2022 cohort of European countries that provided individual data to the ERA Registry. **Source:** Boenink et al. CKJ 2024, https://doi.org/10.1093/ckj/sfae405, Figure 15. The figure was slightly adapted from the original figure (including numbers expressing the reduction in life expectancy in years when compared to the general population). **Explanation:** In the period from 2018 to 2022, the expectancy remaining years of life for all age groups combined was approximately 66% and 68% shorter for males and females on dialysis, and 46% and 49% shorter for males and females living with a functioning kidney graft when compared to males and females in the general population.

